# PPAR-****γ**** Regulates Trophoblast Differentiation in the BeWo Cell Model

**DOI:** 10.1155/2014/637251

**Published:** 2014-02-23

**Authors:** Khrystyna Levytska, Sascha Drewlo, Dora Baczyk, John Kingdom

**Affiliations:** ^1^Research Centre for Women's and Infants' Health, Lunenfeld-Tanenbaum Research Institute, Mount Sinai Hospital, 600 University Avenue, Room 3-904, Toronto, ON, Canada M5G 1Z5; ^2^Department of Laboratory Medicine and Pathobiology, University of Toronto, ON, Canada; ^3^Department of Obstetrics and Gynecology, C. S. Mott Center for Human Growth and Development, Wayne State University School of Medicine, Detroit, MI, USA; ^4^Maternal-Fetal Medicine Division, Department of Obstetrics and Gynecology, Mount Sinai Hospital, 600 University Avenue, Room 3-904, Toronto, ON, Canada M5G 1Z5; ^5^Department of Obstetrics and Gynecology, University of Toronto, Toronto, ON, Canada

## Abstract

Common pregnancy complications, such as severe preeclampsia and intrauterine growth restriction, disrupt pregnancy progression and impair maternal and fetal wellbeing. Placentas from such pregnancies exhibit lesions principally within the syncytiotrophoblast (SCT), a layer in direct contact with maternal blood. In humans and mice, glial cell missing-1 (GCM-1) promotes differentiation of underlying cytotrophoblast cells into the outer SCT layer. GCM-1 may be regulated by the transcription factor peroxisome proliferator-activated receptor-gamma (PPAR-**γ**); in mice, PPAR-**γ** promotes labyrinthine trophoblast differentiation via Gcm-1, and, as we previously demonstrated, PPAR-**γ** activation ameliorates disease features in rat model of preeclampsia. Here, we aimed to characterize the baseline activity of PPAR-**γ** in the human choriocarcinoma BeWo cell line that mimics SCT formation *in vitro* and modulate PPAR-**γ** activity to study its effects on cell proliferation versus differentiation. We report a novel negative autoregulatory mechanism between PPAR-**γ** activity and expression and show that blocking PPAR-**γ** activity induces cell proliferation at the expense of differentiation, while these remain unaltered following treatment with the agonist rosiglitazone. Gaining a deeper understanding of the role and activity of PPAR-**γ** in placental physiology will offer new avenues for the development of secondary prevention and/or treatment options for placentally-mediated pregnancy complications.

## 1. Introduction

During normal pregnancy, the healthy developing placenta ensures effective nourishment of the fetus by facilitating an exchange of gases, nutrients, and waste products between fetal and maternal circulations [1]. The critical cell type in this context is the villous cytotrophoblast (VCT) which constantly forms new syncytiotrophoblast (SCT) throughout pregnancy. VCT cells are a heterogeneous population, comprising of progenitors that divide repeatedly (symmetrically) and others which divide asymmetrically to produce postmitotic cells that develop the potential to fuse into the overlying SCT. The process of cytotrophoblast proliferation, differentiation, and syncytial fusion is required to generate sufficient SCT to cover the developing placental villi [1].

The phenomenon of asymmetric cell division directed by the transcription factor glial cell missing-1 (GCM-1) was first identified in *Drosophila* [2]. In mice, knock-out experiments demonstrated that Gcm-1 is critical for labyrinth formation [3], whereas *Gcm*-1^+/−^ mice are viable with abnormal placental development [4]. Studies of human placental villi report analogous GCM-1 localization and function in villous trophoblast to that seen in rodents [5, 6]. Interestingly, levels of GCM-1 are downregulated in the placentas of women suffering from severe preeclampsia, a pregnancy complication characterized by impaired villous structure and placental development [7, 8].

The transcription factor peroxisome proliferator-activated receptor gamma (PPAR-*γ*) may regulate the process of SCT formation, since it is known to regulate Gcm-1 expression [9, 10] and placental development [11] in mice. The ability to control the transcriptional activity of PPAR-*γ* with highly specific drugs, including the agonist rosiglitazone [12] and the antagonist T0070907 [13] has been utilized in an attempt to study its role in several features of placental development. We have recently shown that rosiglitazone-induced PPAR-*γ* activation is able to ameliorate disease characteristics in the rat model of preeclampsia via its downstream target heme oxygenase-1 (HO-1), an enzyme which produces carbon monoxide (CO, a potent vasodilator) and bilirubin, an antioxidant [14, 15]. Furthermore, other groups have looked at the effect of PPAR-*γ* activity induction on cell migration as well as analyzed expression profiles in VCT and extravillous trophoblast (EVT) cells [10, 16], leading to the conclusion that PPAR-*γ* plays a role in human placental development.

In the present study, we attempted to study the activity of this transcription factor in the human choriocarcinoma-derived cell line BeWo, an established model of SCT formation *in vitro*, and primary trophoblast cells to establish the effect of PPAR-*γ* activity modulation on key features of trophoblast physiology. We hypothesized that stimulating PPAR-*γ* activity with a highly specific agonist rosiglitazone will induce cell differentiation [resulting in increased expression of the differentiation marker GCM-1 and augmented release of cell fusion marker, human chorionic gonadotropin-*β* (*β*-hCG)], while the opposite (i.e., proliferation) will be favored by the antagonist T0070907. To our surprise, when examining the response of *GCM-1* expression and *β*-hCG release, we show that the baseline PPAR-*γ* activity in the BeWo cell line is relatively high, limiting our ability to stimulate it further with the agonist rosiglitazone, but presenting an opportunity to block it with the antagonist T0070907. Interestingly, our ability to stimulate the *GCM-1* response was augmented when the endogenous levels of PPAR-*γ* are downregulated using siRNA, adding support to the concept that PPAR-*γ* regulates the differentiation axis in the BeWo cell line. Although these findings outline the limitation of this cell line, they nonetheless support the wide used of this model in the study of molecular mechanisms present in the human placenta, since the responses of target genes in isolated human cytotrophoblast cells were found to be analogous to those in BeWo cells.

## 2. Materials and Methods

### 2.1. BeWo Cells

The human choriocarcinoma-derived BeWo cell line was purchased from ATCC (Burlington, ON, Canada) and fingerprinted at the Centre for Applied Genomics (SickKids, Toronto, ON, Canada); markers were found to be identical to those in the ATCC database. BeWo cells between passages 10 and 20 were used. For all treatments, cells were maintained in F12K medium (Wisent Inc., St. Bruno, QC, Canada), supplemented with 10% fetal bovine serum (FBS; Canadian grade, heat-inactivated, Invitrogen, Burlington, ON, Canada), 100 units/mL penicillin, 100 µg/mL streptomycin, and 2 nM L-glutamine (Life Technologies, Burlington, ON, Canada), in atmospheric O_2_/5% CO_2_ at 37°C.

For treatments, BeWo cells were seeded at 50,000 cells per 1 mL of media and allowed to attach for 24 hours. The following day, cells were pretreated with the PPAR-*γ* antagonist T0070907 (Cayman Chemical, Ann Arbor, MI, USA) for 30 minutes and were then treated with either the PPAR-*γ* agonist rosiglitazone (Enzo Life Sciences, Burlington, ON, Canada), the antagonist T0070907 (Cayman Chemical), and/or the weak agonist forskolin (Sigma-Aldrich, Oakville, ON, Canada). Cell viability under all treatments was assessed at 48 hours of culture using CytoTox-ONE Homogeneous Membrane Integrity Assay (Promega, Madison, WI, USA). No drug treatments resulted in significant cell toxicity at 48 hours (data not shown).

Following treatment, cells were washed in ice-cold D-PBS (Wisent Inc.) and collected according to different downstream applications. For RNA analysis, cells were collected into RLT Plus buffer (Qiagen, Toronto, ON, Canada) with 10% *β*-mercaptoethanol (Fisher Scientific, Ottawa, ON, Canada). For protein analysis, cells were scraped and collected in RIPA Buffer (Thermo Scientific, Ottawa, ON, Canada) with phosphatase (Phosphatase Inhibitor Cocktail 2, Sigma-Aldrich) and protease inhibitors (Complete Mini, EDTA-free Protease Inhibitor Cocktail Tablets, Roche Applied Science, Laval, QC, Canada). Conditioned medium was collected and centrifuged for 5 minutes at 425 g at room temperature (RT) to remove cellular debris. All collected samples were stored at −80°C for further analysis.

### 2.2. Transfection of BeWo Cells

The BeWo cells were transfected with commercially-available double-stranded siRNA oligonucleotides against the human *PPAR-*γ** sequence (SantaCruz Biotechnology, Dallas, TX, USA). Nonsilencing control (sequence: 5′-TTCTCCGAACGTGTCACGT-3′) was used as a negative control. BeWo cells were plated into 12-well plates and transfected the following day according to the manufacturer's specifications. Briefly, 50%-confluent BeWo cells were transfected with 30 µM of *PPAR-*γ**-targeted siRNA or nonsilencing control with 2.5 µL of PepMute siRNA transfection reagent (SignaGen Laboratories, Ijamsville, MD) per well. The following day, media were changed and cells were treated for another 48 hours. Experiments with fluorescent-labeled siRNA established 80–90% transfection efficiency (data not shown). Toxicity of siRNA treatment was monitored with Human Interferon Alpha ELISA kit (PBL Biomedical Laboratories, Brussels, Belgium).

### 2.3. Human Primary Cytotrophoblast Cell Isolation

First and second trimester (12–19 weeks) human placental tissue was used for the isolation of primary cytotrophoblast (CT) cells. Placentas were obtained from the Morgentaler Clinic (Toronto, Canada) following elective legal terminations of pregnancy. Mount Sinai Hospital Research Ethics Board approval (MSH REB no. 04-0018-U) was obtained and, prior to tissue collection, all patients gave written informed consent; gestational age and viability were established preoperatively. Primary CT cells were isolated according to the following protocol (modified from the original Kliman method [17]). Briefly, placental tissue was separated from membranes and subjected to a 40-min digestion in Trypsin Digestion Cocktail [25 mM Hepes (Sigma-Aldrich), 50 units/mL DNase I (Sigma-Aldrich), 4.25 mM MgSO_4_, 0.125% Trypsin, 2.5 µg/mL fungizone, 100 µg/mL gentamacin (all from Invitrogen), and diluted in 1 : 1 mix of Hank's buffered salt solution (HBSS) with and without Mg^2+^ and Ca^2+^] to remove the SCT layer. Four 20-min digestions were then performed to collect subsequent layers containing primary cytotrophoblast, fibroblast, and endothelial cells. Collected cells were washed and spun in Ficoll-Paque PLUS reagent (1.077 g/mL, GE Healthcare Life Sciences, Sweden) for 10 minutes at 2,000 rpm without a break to remove red blood cells and cell debris. Following separation from red blood cells, the resulting ring of cells was collected, washed, and resuspended in DMEM/F12 medium (Life Technologies), supplemented with 10% FBS (Invitrogen), 100 units/mL penicillin, 100 µg/mL streptomycin, 2 nM L-glutamine (Life Technologies), and 2.5 µg/mL fungizone (Invitrogen). All isolated cells were seeded in 6-well plates at 50% confluency and maintained in cell medium (described above) in 8% pO_2_ at 37°C. The following day, cells were washed once with D-PBS (Wisent Inc.) to remove cellular debris and cultured for an additional 24 hours under different drug treatments or vehicle controls. Cells were collected for RNA analysis according to the protocol described above.

### 2.4. RNA Extraction, Reverse Transcription, and qRT-PCR

BeWo and CT cell RNA was extracted using the RNeasy *Plus* Mini Kit (Qiagen) according to manufacturer's instructions. Five hundred nanograms (ng) of RNA was reverse transcribed using iScript Reverse Transcription Supermix (Bio-Rad, Mississauga, ON, Canada) according to the following protocol: 5 minutes at 25°C, 30 minutes at 42°C, and followed by 5 minutes at 85°C. Gene expression was measured using quantitative real-time PCR (qRT-PCR) and run on the CFX384 Real-Time PCR Detection System (Bio-Rad) with LuminoCt SYBR Green qPCR ReadyMix (Sigma-Aldrich). qRT-PCR reactions were performed according to the following protocol: initial activation at 95°C for 5 minutes, followed by 38 thermal cycles of denaturation at 95°C for 5 seconds, and annealing/extension at 60°C for 20 seconds. Gene expression was normalized to the geometric mean of three housekeeping genes (*HPRT*, *TBP*, and *YWHAZ* for BeWo cells; *HPRT*, *GAPDH*, and *YWHAZ* for primary CT cells). Gene of interest expression in each treatment was expressed as fold change relative to its respective vehicle (set as 1). Primer sequences for all genes are listed in Table 1.

### 2.5. Protein Isolation

For protein analyses, BeWo cells were collected in 250 µL of RIPA lysis buffer with phosphatase and protease inhibitors. Samples were homogenized and placed on a nutator for 1 hour at 4°C, after which they were spun at top speed for 10 minutes at 4°C and the supernatant was collected. Protein concentration was measured using Pierce BCA Protein Kit (Thermo Scientific), according to manufacturer's instructions.

### 2.6. Western Blotting

Twenty µg of total protein (diluted in RIPA Buffer with inhibitors, 4X Loading Dye [Invitrogen], and 10% *β*-mercaptoethanol) was electrophoresed in 1x TG-SDS Buffer (Wisent Inc.) at 50–100 V on 4–20% Mini-PROTEAN TGX Gels (Bio-Rad). Following electrophoresis, proteins were transferred onto PVDF membrane (Bio-Rad) using the Trans-Blot Turbo Transfer System (Bio-Rad). Immediately following the transfer, membranes were blocked in 5% milk/TBS-T (Blotting-Grade Blocker, Bio-Rad; TWEEN 20, Sigma-Aldrich) for 1 hour at RT. All primary antibodies (See Table 1 in Supplementary Material available online at http://dx.doi.org/10.1155/2014/637251) were applied overnight at 4°C in 5% milk/TBS-T. The following day, membranes were washed with 0.001% TBS-T (3 × 20 min). Membranes were incubated for 1 hour at RT with respective secondary antibodies (GE Healthcare UK Limited, UK) diluted 1 : 3,000 in 5% milk/TBS-T. Following washing, membranes were developed using Western Lightning *Plus*-ECL (Thermo Scientific) on Premium Autoradiography Film (Denville Scientific, South Plainfield, NJ, USA). Band intensities within linear range were quantified using Quantity One software (Bio-Rad). Protein levels were normalized to a housekeeping protein (*β*-actin, *α*-tubulin, or lamin B). Protein expression for each condition was further compared to its respective vehicle control (set as 1).

### 2.7. Cellular Fractionation Analysis

Cellular fractionation was performed using the Nuclear Extract Kit (Active Motif, Burlington, ON, Canada), according to manufacturer's protocol. Protein concentration was measured using the Pierce BCA Protein Kit (Thermo Scientific). Protein amount was normalized and prepared for Western blotting for PPAR-*γ*, *α*-tubulin (cytoplasmic protein) and lamin B (nuclear protein). Both fractions were controlled for purity of separation by measuring the expression of the housekeeping protein from the other cellular compartment.

### 2.8. Enzyme-Linked Immunosorbent Assay (ELISA)

Free *β*-hCG ELISA Kit (Phoenix Pharmaceuticals, Inc., Burlingame, CA, USA) was used to measure *β*-hCG release, according to manufacturer's instructions. *β*-hCG protein concentration was quantified using the standard curve and protein levels were normalized to total released protein. *β*-hCG release under different treatments was further normalized to respective vehicle controls (set as 1).

### 2.9. Fluorescent Immunohistochemistry (IHC)

Immunofluorescence was used to visualize PPAR-*γ* protein localization and BeWo cell fusion. The following protocol was used for the experiments. Coverslips were submerged into 100% ethanol and left to dry under UV light for 30 minutes. BeWo cells were plated on coverslips at a density of 150,000 cells per well in 6-well plates. Cells were incubated with treatments outlined above. Following treatment, cells were washed in ice-cold D-PBS (Wisent Inc.), fixed in 1 : 1 methanol : acetone solution for 3 minutes on ice, and washed with D-PBS. Cells were permeabilized in 0.2% TritonX-100 (Fisher Scientific) for 5 minutes on ice, washed, and blocked for 1 hour at RT in Protein Block Serum-Free, Ready-To-Use (DAKO, Carpinteria, CA, USA). Primary antibodies (Supplementary Table 1) were diluted in blocking solution and incubated overnight at 4°C. The following day, the PPAR-*γ* signal was amplified with anti-rabbit biotinylated antibody (diluted 1 : 300 in blocking solution) for 1 hour at RT. Lastly, DAPI (1 : 1,000; Sigma-Aldrich), anti-mouse Alexa546 antibody (1 : 200; Invitrogen), and SA-Alexa488 (1 : 1,000; Invitrogen), all diluted in blocking solution, were incubated for 1 hour at RT. Coverslips were mounted on slides using an Immu-Mount mounting medium (Thermo Scientific). Slides were left to dry in the dark and stored at 4°C until further analysis.

Fluorescent microscopy was performed using the Spinning Disc Confocal Microscope (DMI6000B, Leica Microsystems, Concord, ON, Canada). Z stacks were taken using Volocity software, Version 5.3.0 (PerkinElmer, Woodbridge, ON, Canada) and deconvolved using Huygens Essential software, Version 4.2.2 (Scientific Volume Imaging, Hilversum, The Netherlands). All images were taken on the same day, under the same acquisition settings, normalized to the highest PPAR-*γ* expression under T0070907 treatment, to minimize variability and allow parallel comparison in protein expression across time points and treatments. For fusion visualization, images were taken at 200x magnification; for PPAR-*γ* localization analysis, Z stacks were taken at 630x magnification.

### 2.10. Luciferase Assay

One kb region upstream of the human *GCM-1* promoter was analyzed for putative PPAR-*γ* binding sequences using the Gene2Promoter software (Version 6.3, Genomix, Germany; Supplementary Figure 1(a)); two putative binding sequences were identified. Oligonucleotides of these sites were synthesised and linkers added for cloning into the pGL4.10[*luc2*] cloning vector (binding sequences 1 and 2; Promega). Sites were mutated to generate sequence-based controls (mutated sequences 1 and 2). Binding and mutated sequences are outlined in Supplementary Figure 1(b). Oligonucleotides and vectors were digested independently, purified and ligated. One-Shot TOP10 Chemically Competent *E*. *coli* cells (Invitrogen) were transformed and plated on ampicillin-positive plates. Antibiotic-resistant clones were isolated using Plasmid *Plus* Midi Kit (Qiagen), sequenced to confirm insertion, and brought to similar concentrations. For transfection experiments, BeWo cells were seeded in tissue culture-suitable 96-well plates (Greiner bio-one, Monroe, NC, USA) at 12,500 cells/well to obtain 30% confluency. The following day, cells were transfected using ExGene 500 transfection reagent (Fermentas, Pittsburgh, PA, USA); efficiency was confirmed using a GFP-expressing control plasmid (data not shown). Transfection optimization experiments established that 200–300 ng of plasmid DNA were optimal for 70–90% transfection efficiency using 0.7 µL of transfection reagent. Generated transfection mixes (for binding and mutated sequences) contained a 10 : 1 composition of experimental vector to a coreporter vector (*renilla*). Following their application, culture plates were centrifuged for 5 minutes at 300 g, according to the manufacturer's protocol. Eight hours following transfection, cells were treated for 24 hours, following which media were removed and cells were lysed with Passive Lysis Buffer, according to Dual-Luciferase Reporter Assay System protocol (Promega). Luciferase activity was measured using an automated photometer. *Renilla* luciferase activity was an internal calibrator; relative luciferase activity with each plasmid (binding sequences 1 and 2) and drug was normalized to its corresponding vehicle control. Furthermore, to reduce random allosteric sequence-dependent background signal, a signal obtained with each binding sequence was normalized to the corresponding mutated sequence.

### 2.11. Cell Proliferation Assay

Relative BeWo cell numbers were assessed using the CellTiter-Fluor Cell Viability Assay (Promega). In 96-well plates (Greiner bio-one), cells were seeded at 10,000 cells/well and treated the following day with drugs as described above. Cell number was measured at 48 hours of culture according to the manufacturer's protocol. The plate was read using the spectrometer and recorded OD values were blanked using the media-only control to account for background fluorescence. Fluorescent measurements obtained with this assay represented an indication of the relative BeWo cell number, such that cell proliferation could be assessed between different drug treatments relative to their respective controls (set as 1).

### 2.12. Statistical Analysis

Experiments were performed in technical duplicates of at least three biological replicates. Data are represented as mean ± standard error of the mean (SEM). Student's *t*-test was used to make comparison drug treatments to corresponding vehicle controls. One-way ANOVA followed by the Newman-Keuls Multiple Comparison Test was used to compare between treatment groups. All statistical calculations were performed using GraphPad Prism 5.2 software. *P* values < 0.05 were considered significant.

## 3. Results

### 3.1. PPAR-*γ* Expression Inversely Correlates with Its Activity

**Figure 1:**
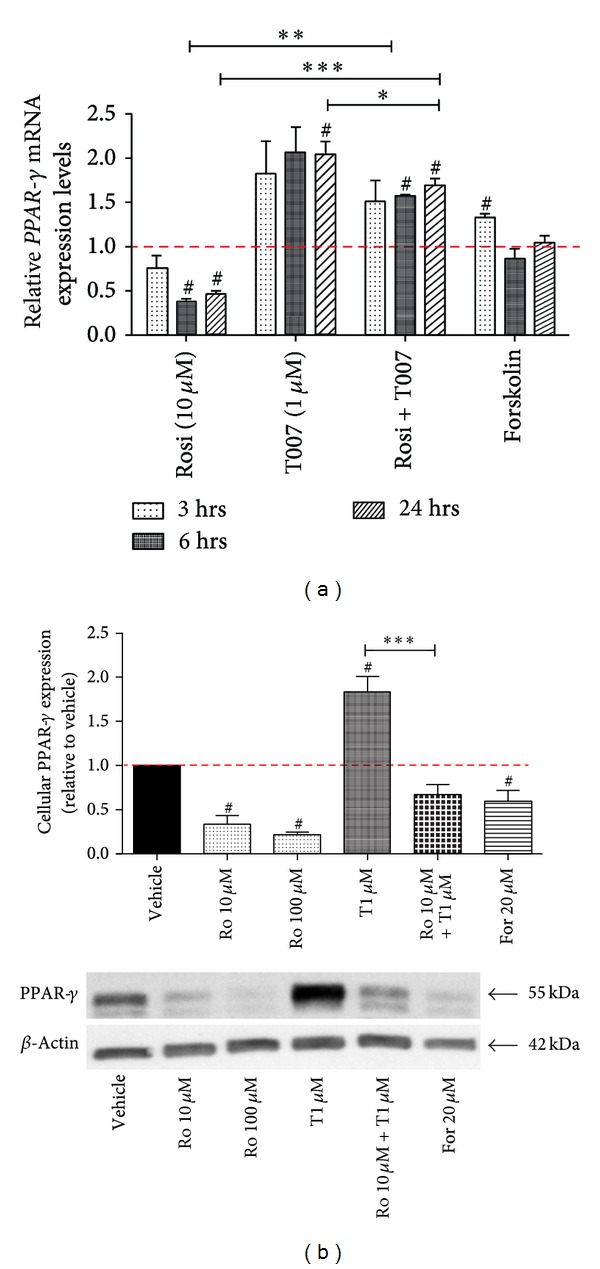
PPAR-*γ* expression correlates inversely with its activity. (a) *PPAR*-*γ* mRNA expression levels overtime in response to rosiglitazone, T0070907, their combination, and forskolin treatment. (b) Cellular PPAR-*γ* protein expression at 48 hours of treatment (representative blot in the bottom panel). All treatments are compared to their respective vehicle controls (set as 1, red dashed line). Values are represented as mean ± SEM; ^#^ *P* < 0.05 versus vehicle control; *P < 0.05; **P < 0.005; ***P < 0.001 (*n* = 4). Ro/rosi, rosiglitazone; T/T007, T0070907; For, forskolin.

In the first set of experiments, we attempted to study the response of PPAR-*γ* expression to the modulation of its activity. We assessed the mRNA and protein expression following rosiglitazone (the agonist) and T0070907 (the antagonist) treatment at different time-points (Figure 1). When the mRNA expression profile was assessed at 3, 6, and 24 hours, we found that induction of PPAR-*γ* activity with rosiglitazone resulted in a decrease of receptor expression, while treatment with T0070907 had the opposite effect, with the most pronounced changes observed 24 hours after treatment (Figure 1(a)). Following activation of PPAR-*γ* with rosiglitazone, *PPAR-*γ** mRNA expression decreased significantly to 47 ± 3.4% by 24 hours compared to vehicle control (*P* < 0.05, *n* = 4). Conversely, blocking PPAR-*γ* activity with T0070907 resulted in a significant upregulation of its expression by 24 hours (2.0 ± 0.1-fold, *P* < 0.05). Forskolin alone did not have a significant effect on *PPAR-*γ** expression at 24 hours. Coadministration of T0070907 with rosiglitazone significantly ameliorated rosiglitazone-induced *PPAR-*γ** downregulation at 6 (*P* < 0.01) and 24 (*P* < 0.001) hours of treatment (versus rosiglitazone alone).

Next, we examined cellular PPAR-*γ* protein expression at 48 hours of treatment using Western blotting (Figure 1(b)). Total PPAR-*γ* protein levels were decreased significantly to 34 ± 9.8% and to 22 ± 6.3% following treatment with the lower and higher dose of rosiglitazone, respectively (*P* < 0.05, *n* = 4). On the contrary, PPAR-*γ* expression rose significantly by 1.83 ± 0.18-fold following treatment with T0070907 (*P* < 0.05). PPAR-*γ* levels also decreased significantly following treatment with forskolin, a weak agonist of PPAR-*γ* (*P* < 0.05). Although the combination of rosiglitazone and T0070907 resulted in a downward trend of PPAR-*γ* expression levels when compared to vehicle control, this failed to reach significance (*P* = 0.0623). Based on our mRNA and protein analyses, we observed that PPAR-*γ* expression and activity are inversely related, such that inducing receptor activity results in a downregulation of its expression and *vice versa*.

### 3.2. PPAR-*γ* Protein within the Nuclear Compartment Responds to PPAR-*γ* Activity-Modulating Drugs

**Figure 2:**
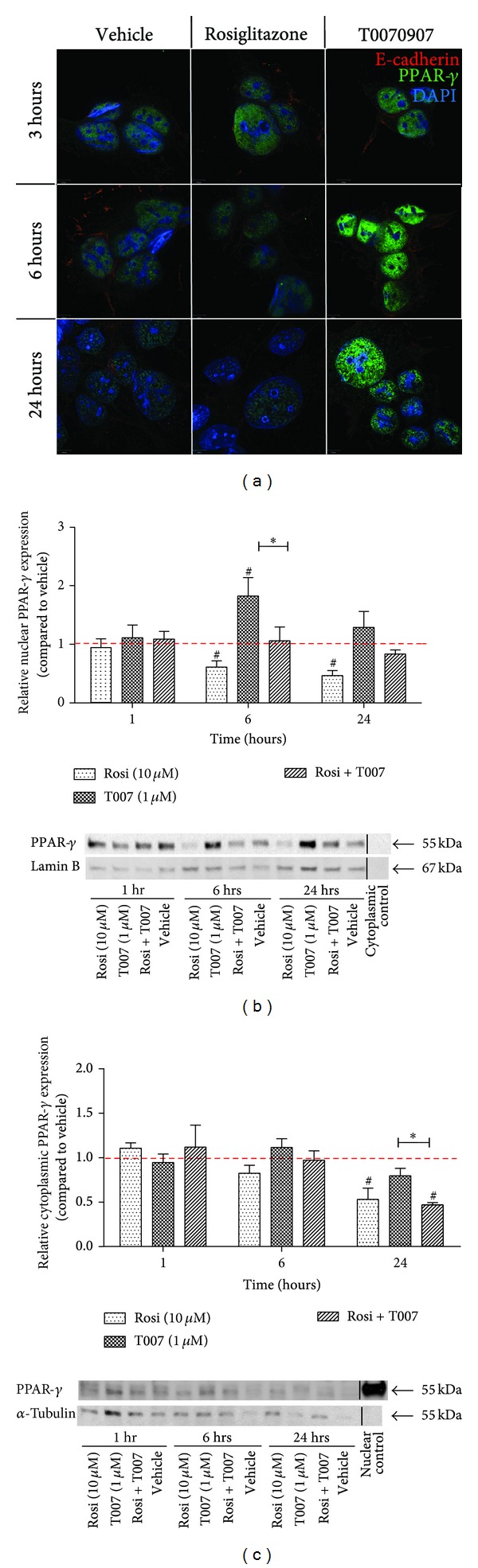
Nuclear, not cytoplasmic, PPAR-*γ* expression changes upon drug treatment. (a) PPAR-*γ* expression was visualized at 3 (top), 6 (middle), and 24 (bottom) hours following treatment with vehicle, rosiglitazone, or T0070907. PPAR-*γ* shown in green, e-cadherin (cell surface marker) in red, DAPI (nuclear marker) in blue; 630X magnification. Nuclear (b) and cytoplasmic (c) PPAR-*γ* protein expression was assessed at 1, 6, and 24 hours of treatment (representative images below). PPAR-*γ* protein levels were assessed using Western blotting; nuclear expression was normalized to lamin B; cytoplasmic expression normalized to *α*-tubulin. Each treatment was further normalized to vehicle (set as 1, red dashed line). Values are represented as mean ± SEM; ^#^ *P* < 0.05 versus vehicle control; *P < 0.05 (*n* = 7–9). Rosi, rosiglitazone; T007, T0070907.

PPAR-*γ* cellular localization and expression was assessed using fluorescent IHC and cellular fractionation analysis. Protein localization was visualized at 3, 6, and 24 hours of treatment with rosiglitazone (10 µM), T0070907 (1 µM), or vehicle (Figure 2(a)). PPAR-*γ* was found to mainly localize in the nucleus with some expression seen in the cytoplasm. Concurrent with the PPAR-*γ* protein analysis using Western blotting, there was an increased expression of PPAR-*γ* after T0070907 treatment and a decrease in staining levels after rosiglitazone treatment.

Cellular fractionation analysis was also used to study changes in PPAR-*γ* protein localization after 1, 6, and 24 hours of treatment (Figures 2(b) and 2(c)). Although no changes in protein expression were seen after 1 hour, following 6 hours of rosiglitazone treatment, nuclear PPAR-*γ* levels decreased significantly by 39 ± 10.8% (*P* < 0.05, *n* = 7–9), while T0070907 treatment led to a significant rise in PPAR-*γ* levels by 1.8 ± 0.3-fold (*P* < 0.05). Combining both drugs did not alter PPAR-*γ* protein expression compared to vehicle (Figure 2(b)). On the contrary, no expression changes were seen in the cytoplasmic fraction at 6 hours of treatment (Figure 2(c)).

PPAR-*γ* expression changes were pronounced in both cellular compartments after 24 hours of treatment. Within the nuclear fraction, the rosiglitazone-induced decrease in protein expression remained significant (down by 53 ± 8.6%, *P* < 0.05), while the effect of T0070907 was less pronounced. Furthermore, at 24 hours of treatment, rosiglitazone led to lower levels of PPAR-*γ* within the cytoplasm (0.5 ± 0.1-fold, *P* < 0.05), as did the combination of rosiglitazone and T0070907 (0.5 ± 0.02-fold versus vehicle, *P* < 0.05). Collectively, our results illustrate that following agonist and/or antagonist treatments, protein expression changes within the cell nucleus were more pronounced compared to changes in the cell cytoplasm.

### 3.3. Blocking PPAR-*γ* Activity Decreases *GCM-1* Transcription

The BeWo cell model was used to study the effect of pharmacological PPAR-*γ* activity modulation on the proliferation/differentiation balance, an important physiologic process within the villous trophoblast layer. We assessed these events by studying markers of syncytial differentiation (such as *GCM-1* expression and free *β*-hCG release) and measuring BeWo cell proliferation.

**Figure 3:**
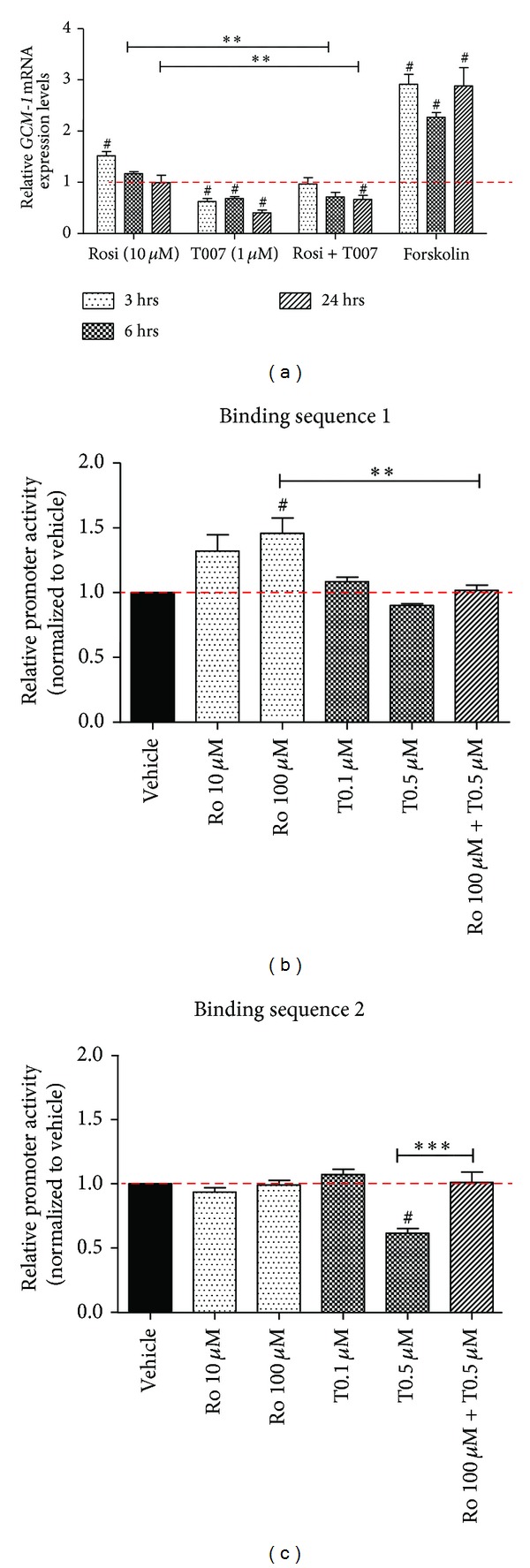
Activity of PPAR-*γ* within the 1kb region of human *GCM*-1 promoter is relatively high. (a) *GCM*-1 expression levels overtime in response to rosiglitazone, T0070907, their combination, and forskolin treatment. PPAR-*γ* activity within the *GCM*-1 promoter [PPAR-*γ* response element 1 (b) and 2 (c)] measured by the luciferase reporter assay at 24 hours of treatment. Treatments are compared to their respective vehicle (set as 1, red dashed line). Values are represented as mean ± SEM; ^#^ *P* < 0.05 versus vehicle control; **P < 0.005; ***P < 0.001 (*n* = 3-4). Ro/rosi, rosiglitazone; T/T007, T0070907.

It has been previously described that lack of Ppar-*γ* results in lower Gcm-1 expression in mouse trophoblast stem cells [9]; therefore, we studied the response of *GCM-1* in BeWo cells to rosiglitazone and T0070907 treatments at 3, 6, and 24 hours of treatment (Figure 3(a)). Activation of PPAR-*γ* with the agonist led to a transient induction of *GCM-1* expression, significant only at 3 hours of treatment (1.5 ± 0.1-fold, *P* < 0.05, *n* = 4). Independent evidence of induction of PPAR-*γ* activity was inferred by observing a robust increase in another PPAR-*γ* target, heme oxygenase-1 (HO-1), at both the mRNA and protein levels (Supplementary Figure 2). Conversely, blocking PPAR-*γ* activity with T0070907 resulted in a prolonged and significant reduction in *GCM-1* mRNA expression (60 ± 5.4% decrease) which persisted at 24 hours (*P* < 0.05). When both rosiglitazone and T0070907 were combined, a significant 34 ± 8.2% reduction in *GCM-1* mRNA expression was observed at 24 hours versus vehicle (*P* < 0.05). Forskolin alone promoted a much greater upregulation of *GCM-1* expression in BeWo cells, evident as early as 3 hours after treatment (2.9 ± 0.2-fold increase, *P* < 0.05); this effect was also significant at 6 (2.3 ± 0.1-fold rise, *P* < 0.05) and 24 hours (2.9 ± 0.4-fold change, *P* < 0.05). Importantly, changes in *GCM-1*, *PPAR-*γ**, and *HO-1* mRNA levels as described thus far were found to be analogous to those in isolated human primary cytotrophoblast cells, supporting the notion that the responses seen in the BeWo cell line are representative of the true human placental phenotype and are neither culture effects nor confined to a cell line (Supplementary Figure 3).

### 3.4. Baseline PPAR-*γ* Activity within the *GCM-1* Promoter is High in BeWo Cells

Despite being able to block the activity of PPAR-*γ* and decrease gene expression of its downstream targets in BeWo cells, it was considerably more challenging to induce its activity above baseline, thereby upregulating the expression of *GCM-1*. Because of this inability to induce *GCM-1* expression over a prolonged period of time, we decided to study the transcriptional activity of PPAR-*γ* in BeWo cells, using the luciferase reporter assay, to test the hypothesis that BeWo cells operate in a state of a high baseline PPAR-*γ* activity (Figures 3(b) and 3(c)). We studied PPAR-*γ* binding to two PPREs (binding sequences 1 and 2) in the upstream 1kb region of the human *GCM-1* gene (see Supplementary Figure 1 for gene map).

Following a 24-hour treatment of BeWo cells with rosiglitazone (10 µM), luciferase activity for binding sequence 1 was increased by 32 ± 12.7%, while failing to reach statistical significance (*P* = 0.1274, *n* = 4; Figure 3(b)). The higher dose of rosiglitazone (100 µM) significantly increased luciferase activity by 46 ± 11.8% (*P* < 0.05), and although there was no response to T0070907 treatment alone, in combination with the higher dose of rosiglitazone, T0070907 significantly blocked the effect of rosiglitazone (*P* < 0.01 versus rosiglitazone alone).

Luciferase activity under the control of binding sequence 2 is shown in Figure 3(c). Here, rosiglitazone treatment did not have an effect on PPAR-*γ* activity, but T0070907 treatment led to a significant 38 ± 3.6% reduction in enzyme activity (*P* < 0.05, *n* = 4). Interestingly, a combination of rosiglitazone and T0070907 significantly increased receptor activity when compared to T0070907 alone (*P* < 0.001) indicating that rosiglitazone was able to exert its effects on transcription.

### 3.5. Lower Endogenous *PPAR-*γ** Levels Lead to a Stronger Rosiglitazone-Induced *GCM-1* Response

**Figure 4:**
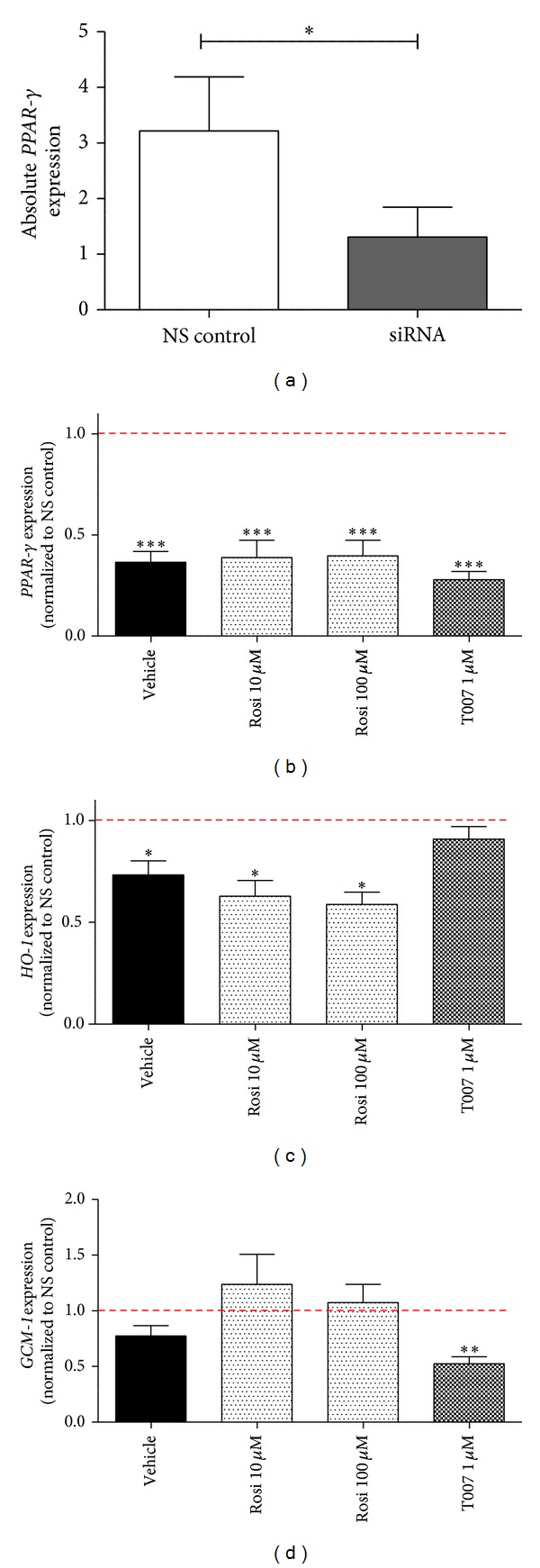
Effect of *PPAR-*γ** downregulation on gene expression following treatment with PPAR-*γ* activity-modulating drugs. (a) *PPAR-*γ** expression was significantly downregulated after 48 hours of treatment with a specific siRNA sequence. *PPAR-*γ** (b), *HO-1* (c), and *GCM-1* (d) expression in the siRNA-treated cells was compared to the same drug treatment in the nonsilencing control-treated cells to elucidate the contribution of PPAR-*γ* to changes in target gene expression. All drug treatments (vehicle, rosiglitazone, and T0070907) under siRNA treatment were normalized to the same treatment under nonsilencing control (red dashed line, set as 1). Values are represented as mean ± SEM; *P < 0.05, **P < 0.005, ***P < 0.001, ****P < 0.0001 (*n* = 4). NS, nonsilencing; rosi, rosiglitazone; T007, T0070907.

To assess whether the changes in levels of target genes were PPAR-*γ*-specific and not artifacts of cell culture, we performed a series of *PPAR-*γ** silencing experiments (Figure 4). Using siRNA oligonucleotides targeted against the human *PPAR-*γ** gene, we successfully downregulated the expression of *PPAR-*γ** by 63 ± 5.3% (*P* = 0.0003, *n* = 4; Figure 4(a)). Interestingly, when drug treatments were compared to vehicle controls within respective siRNA-treated and nonsilencing control-treated groups, the gene expression profiles were analogous in both conditions, indicating that the system remained functional overall, regardless of whether the levels of *PPAR-*γ** were significantly downregulated or not (data not shown).

To elucidate the contribution of PPAR-*γ* itself, the target gene mRNA expression in response to each drug in the siRNA-treated sample was compared to the same drug treatment in the nonsilencing (ns) control-treated sample (ns control was set as 1). Such an approach allowed us to calculate whether, and by how much, the mRNA responses were ameliorated or augmented by the downregulation of endogenous *PPAR-*γ** levels. Together, our findings show that the responses seen following agonist and antagonist treatment are indeed PPAR-*γ*-dependent.

First, we studied the expression of PPAR-*γ* under such conditions (Figure 4(b)). As predicted, the expression of PPAR-*γ* was significantly decreased under all treatments (vehicle, rosiglitazone, and T0070907) in the siRNA-treated group when compared to the nonsilencing controls (*P* < 0.001; *n* = 4). Next, we examined the effect of PPAR-*γ* downregulation on expression levels of a potent and easily-inducible downstream target, HO-1. mRNA analysis supported the notion that responses of *HO-1* to drugs are PPAR-*γ*-dependent, such that *PPAR-*γ** downregulation resulted in lower levels of *HO-1* transcription under vehicle control condition and both doses of rosiglitazone (Figure 4(c)). Under baseline (i.e., vehicle) conditions, PPAR-*γ* downregulation led to a significant 27 ± 6.8% decrease in *HO-1* levels, while treatment with lower and higher doses of rosiglitazone ameliorated *HO-1* response by 38 ± 7.7% and 42 ± 6.1%, respectively, when compared to analogous treatments in the non-silenced group (*P* < 0.05, *n* = 4). Response to T0070907 did not differ between siRNA-treated and non-treated cells.

Lastly, we analyzed the response of *GCM-1* transcription to *PPAR-*γ** downregulation and treatment with PPAR-*γ* activity-modulating drugs (Figure 4(d)). Our findings illustrate that *GCM-1* response to rosiglitazone is augmented by siRNA treatment compared to the same drug treatment in the non-silenced group at 48 hours. By decreasing endogenous *PPAR-*γ** levels, we observed a trend towards a stronger *GCM-1* response with the lower dose of rosiglitazone, as well as a significant reduction of *GCM-1* expression under T0070907 treatment (*P* < 0.005, *n* = 4). Such findings support our observations of high baseline PPAR-*γ* transcriptional activity within the *GCM-1* promoter region assessed by the luciferase reporter assay (described in Figures 3(b) and 3(c)).

### 3.6. Blocking PPAR-*γ* Activity Inhibits Differentiation and Induces Proliferation of BeWo Cells

**Figure 5:**
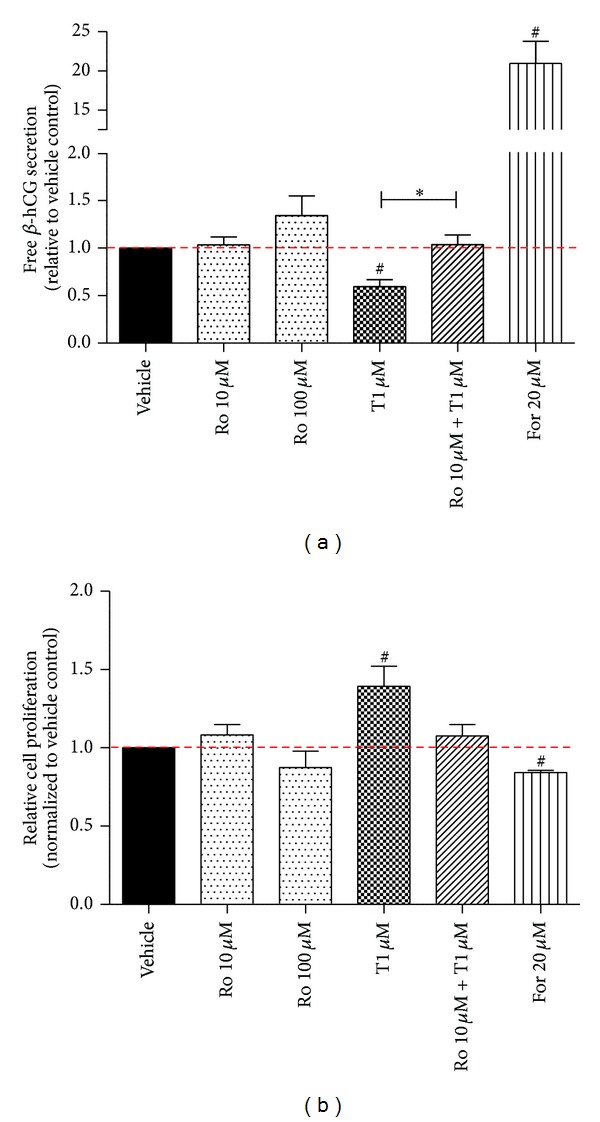
Effect of PPAR-*γ* manipulation on free *β*-hCG release from BeWo cells and cell number. (a) Free *β*-hCG release was measured at 48 hours after-treatment and its levels were normalized to total protein content in the media. (b) BeWo cell numbers were measured using CellTiter-Fluo Cell Viability Assay at 48 hours of treatment. Each drug treatment was compared to its respective vehicle control and expressed as fold-change (vehicle set as 1, red dashed line). Values are represented as mean ± SEM; ^#^ *P* < 0.05 versus vehicle control; *P < 0.05; (*n* = 4–7). Ro: rosiglitazone; T: T0070907; For: forskolin.

To complete the study of the role of PPAR-*γ* on the maintenance of proliferation/differentiation balance, we assessed differentiation of BeWo cells using free *β*-hCG as a marker (Figure 5(a)). Furthermore, we assessed cell fusion morphologically using e-cadherin as a cell surface marker (Supplementary Figure 4). In our experiments, forskolin, a known inducer of free *β*-hCG secretion [18], was used as a positive control for cell differentiation. A 20.9 ± 2.8-fold induction in *β*-hCG release following forskolin treatment was seen at 48 hours (*P* < 0.05, *n* = 7). Lower dose of rosiglitazone (10 µM) did not affect free *β*-hCG release, while a higher concentration of the drug showed only an upward trend in *β*-hCG release (34 ± 20.4% increase, *P* = 0.19, *n* = 4). On the contrary, blocking PPAR-*γ* activity with T0070907 significantly downregulated *β*-hCG secretion by 41 ± 7.3% (*P* < 0.05, *n* = 7). Coadministration of both the inhibitor and the activator resulted in no change of *β*-hCG protein release compared to vehicle.

The effect of PPAR-*γ* activity modulation on the BeWo cell number was assessed after 48 hours of treatment (Figure 5(b)). Forskolin treatment, which is known to induce differentiation and decrease cell proliferation [5], significantly decreased BeWo cell numbers at 48 hours by 16 ± 1.4% (*P* < 0.05, *n* = 4). Treatment of BeWo cells with T0070907 significantly increased BeWo cell numbers by 39 ± 12.8% when compared to vehicle control (*P* < 0.05). Rosiglitazone did not have an effect on the BeWo cell number as predicted.

## 4. Discussion

The present study has established the regulatory role of PPAR-*γ* on the differentiation of the villous trophoblast layer, represented by the BeWo cell line model of SCT formation *in vitro*, together with supporting evidence from primary isolated human cytotrophoblast cells. We report a novel finding of a negative autoregulatory mechanism of PPAR-*γ* expression in response to its activity modulation with the agonist rosiglitazone and the antagonist T0070907. Furthermore, we show the role of PPAR-*γ* in the differentiation of BeWo cells into confluent syncytialized structures that mimic the SCT layer *in vivo*. Importantly, these findings are analogous to those found in isolated human primary CT cells suggesting that the BeWo cell line represents molecular mechanisms within the human placenta. In our BeWo model, blocking PPAR-*γ* activity with an antagonist T0070907 promoted cell proliferation at the expense of fusion, reflected by a decrease in both mRNA expression of the transcription factor *GCM-1* and free *β*-hCG release into the overlying media. Conversely, induction of PPAR-*γ* activity in BeWo cells with the agonist rosiglitazone did not produce the opposite effects, suggesting that PPAR-*γ* activity is maximal in BeWo cells to drive the process of syncytialization, although induction of this activity is possible in a different pathway, as demonstrated by a rise in another target gene (HO-1) expression. Furthermore, this effect was found to be PPAR-*γ*-specific, such that these responses were partially ameliorated by the downregulation of endogenous *PPAR-*γ** levels.

We tested the hypothesis that PPAR-*γ* activity is modulated via a negative feedback loop. We observed that PPAR-*γ* participates in a negative autoregulation feedback mechanism, whereby the induction of PPAR-*γ* activity is accompanied by a decrease in its expression, while the opposite occurs following treatment with the antagonist. This suggests that certain molecular mechanisms in the BeWo cell line are in place to ensure fine-tuning of PPAR-*γ* activity: induction of activity is compensated for by a decrease in transcription factor expression, while a decrease in its activity is complimented with a rise in receptor levels. Although it has been reported that PPAR-*γ* cofactors (such as PPAR-*γ* Coactivator-1*β*) [19, 20] and coreceptors (such as Liver X Receptor-*α*) [21] participate in positive autoregulatory loops, simultaneous findings by us and Knabl et al. [22] are the first reports to show negative PPAR-*γ* autoregulation in the human cell line BeWo. This has important implications, considering that PPAR-*γ* is a transcription factor with an array of functions and plays a role in lipid metabolism, cell growth, differentiation, and so forth; carefully regulating its activity is crucial for cellular hemostasis and timely cell cycle progression.

We further analyzed PPAR-*γ* expression changes in both cellular compartments (nucleus and the cytoplasm) following drug treatments. We observed that nuclear fluctuations in receptor levels were more robust when cells were treated with the agonist and the antagonist, while changes in the cytoplasmic compartment were more subtle and delayed. This suggests that changes in the nuclear compartment, consistent with its primary action as a nuclear receptor and transcription factor, are mostly responsible for fluctuations seen in whole cell lysates. These changes were confirmed using fluorescent immunohistochemistry assessments, and, thus, were not an artifact of the cellular fractionation technique caused by leaking or active transport of PPAR-*γ* out of the nucleus upon cell lysis. This finding leads us to speculate that changes in the nucleus may be due to altered protein stability (possibly affected by ubiquitination or unfolded protein response) or shunting between the two cell compartments following drug treatment. Collectively, our findings suggest that there are differential PPAR-*γ* protein regulation mechanisms in the nucleus and the cytoplasm.

Furthermore, we studied the effect of PPAR-*γ* activity modulation on the balance between continued BeWo cell proliferation, as opposed to the commitment to terminal differentiation via syncytialization, by monitoring the expression of differentiation-promoting transcription factor GCM-1, together with the release of free *β*-hCG into the overlying media. To our surprise, we found that attempts to stimulate PPAR-*γ* with rosiglitazone did not induce *GCM-1* expression beyond 3 hours of treatment and did not result in a rise in *β*-hCG release. This was true despite both *GCM-1* induction and a rise of free *β*-hCG secretion by forskolin treatment and possibility of stimulation of PPAR-*γ* transcriptional activity as seen by a robust rise in levels of another target, HO-1, following rosiglitazone treatment. By contrast, the antagonist T0070907 had a pronounced effect causing a significant reduction in both *GCM-1* expression and *β*-hCG release; this suppression of differentiation was accompanied by a predicted rise in cell proliferation, indicating that the antagonist was able to exert a strong repressive effect on the PPAR-*γ*/GCM-1/syncytialization axis. We found a plausible explanation for this observation using the luciferase reporter system, whereby we observed that rosiglitazone treatment could not further stimulate PPAR-*γ* binding to the *GCM-1* promoter whereas the antagonist T0070907 significantly decreased the interaction between PPAR-*γ* and its response element. Downregulation of *PPAR-*γ** expression using siRNA oligonucleotides further supported this observation as *GCM-1* rise due to rosiglitazone treatment was augmented under lower endogenous *PPAR-*γ** levels. Although these results did not reach statistical significance, they may be explained by the incomplete receptor knockdown as well as the presence of the autoregulatory PPAR-*γ* feedback mechanism. Collectively, these findings suggest that the baseline activity of PPAR-*γ* in the BeWo cell line is relatively high, limiting the potential for its further induction with an agonist treatment.

Our studies support a role for PPAR-*γ* in mediating key functions of the trophoblast layer that is in direct contact with maternal blood. In this location, PPAR-*γ* may potentially navigate the balance between the need to retain a proliferating population of CT lineage-restricted progenitors with the need to constantly form an overlying SCT layer. The majority of pregnancy complications requiring preterm delivery, mainly severe preeclampsia and intrauterine growth restriction, exhibit structural abnormalities of placental villi [8, 23], including increased apoptosis of the villous trophoblast compartment [24], depletion of proliferating CT cells and patchy areas of apoptosis and necrosis in the SCT layer [25]. Our capacity to pharmacologically induce PPAR-*γ* activity poses a potential avenue for improving placental trophoblast physiology via upregulation of GCM-1 expression in residual trophoblast progenitors, thereby restoring the process of syncytiotrophoblast formation, and thus normal placental function, in pregnancies characterized by abnormal placental development.

## 5. Conclusions

PPAR-*γ* is one of key metabolic regulators in the human body and has recently been suggested to play a role in physiologic placental development and, thus, normal pregnancy progression. Its role has been implicated in several pregnancy complications, including preeclampsia [14, 26], gestational diabetes [22], and hypoxia-induced fetal growth restriction at high altitudes [27]. Elucidating the role of this receptor in human trophoblast cell lineage differentiation and healthy placentation is instrumental to the utilization of these pathways for advancement of therapeutics. Our ability to pharmacologically manipulate this metabolic regulator is an invaluable tool for the development of possible prophylactic and/or treatment options for women at risk of developing and suffering from common pregnancy complications.

## Supplementary Material

Supplementary Material contains information which supports or further explains the findings of this manuscript. Included in this report are the *Methods* table (Supplementary Table 1) and figure (Supplementary Figure 1) outlining antibodies utilized for this study and plasmid design, respectively; as well as the *Results* supporting figures, specifically: findings supporting our ability of induction of PPAR-*γ* transcriptional activity via HO-1 as a downstream target (Supplementary Figure 2); the response of primary cytotrophoblast cells to PPAR-**γ** activity-modulating drugs (Supplementary Figure 3); and the morphological assessment of BeWo cell fusion in response to these treatments (Supplementary Figure 4).

## Figures and Tables

**Table 1:** Primer sequences.

Gene	Primer sequence (5′ → 3′)		Number of bases	Primer pair efficiency
*GCM-1 *	Forward	ATG GCA CCT CTA GCC CCT ACA	21	102.5%
	Reverse	GCT CTT CTT GCC TCA GCT TCT AA	23	
*PPAR-*γ**	Forward	CTC AGT GGA GAC CGC CCA GG	20	109.2%
	Reverse	GCT CCA GGG CTT GTA GCA GG	20	
*HMOX-1 *	Forward	CGG CTT CAA GCT GGT GAT GGC	21	110.6%
	Reverse	CCT GCT CCA GGG CAG CCT TG	20	
*GAPDH *	Forward	AGA TCA TCA GCA ATG CCT CC	20	108.2%
	Reverse	CAT GAG TCC TCC CAC GAT AC	20	
*YWHAZ *	Forward	ACT TTT GGT ACA TTG TGG CTT CAA	24	95.3%
	Reverse	CCG CCA GGA CAA ACC AGT AT	20	
*TBP *	Forward	TGC ACA GGA GCC AAG AGT GAA	21	110.4%
	Reverse	CAC ATC ACA GCT CCC CAC CA	20	
*HPRT *	Forward	TGA CAC TGG CAA AAC AAT GCA	21	95.7%
	Reverse	GGT CCT TTT CAC CAG CAA GCT	21	
