# Supplementary material for: PPAR-γ Regulates Trophoblast Differentiation in the BeWo Cell Model
Source: PPAR Res. 2014 Feb 23;2014:637251. doi: 10.1155/2014/637251 (PMC3953466; doi:10.1155/2014/637251)
Supplement: Supplementary file 1 — Supplementary Material contains information which supports or further explains the findings of this manuscript. Included in this report are the Methods table (Supplementary Table 1) and figure (Supplementary Figure 1) outlining antibodies utilized for this study and plasmid design, respectively; as well as the Results supporting figures, specifically: findings supporting our ability of induction of PPAR-γ transcriptional activity via HO-1 as a downstream target (Supplementary Figure 2); the response of primary cytotrophoblast cells to PPAR-γ activity-modulating drugs (Supplementary Figure 3); and the morphological assessment of BeWo cell fusion in response to these treatments (Supplementary Figure 4). [file 637251.f1.zip › 637251.f1/mat.637251.v1.pdf]

Supplementary Table 1. Antibodies and dilutions.

Antigen	Origin	Size (kDa)	Dilution	Supplier
HO-1	Rabbit	32	1:500 (WB)	Enzo Life Sciences<br>Burlington, ON, Canada
PPAR-γ	Rabbit	54-57	1:100 (WB)<br>1:100 (F-IHC)	SantaCruz Biotechnology<br>Dallas, TX, USA
E-cadherin	Mouse	110	1:1000 (WB)<br>1:100 (F-IHC)	Abcam<br>Toronto, ON, Canada
Lamin B	Mouse	67	1:500 (WB)	SantaCruz Biotechnology<br>Dallas, TX, USA
α-Tubulin	Mouse	50	1:2000 (WB)	Sigma<br>Oakville, ON, Canada

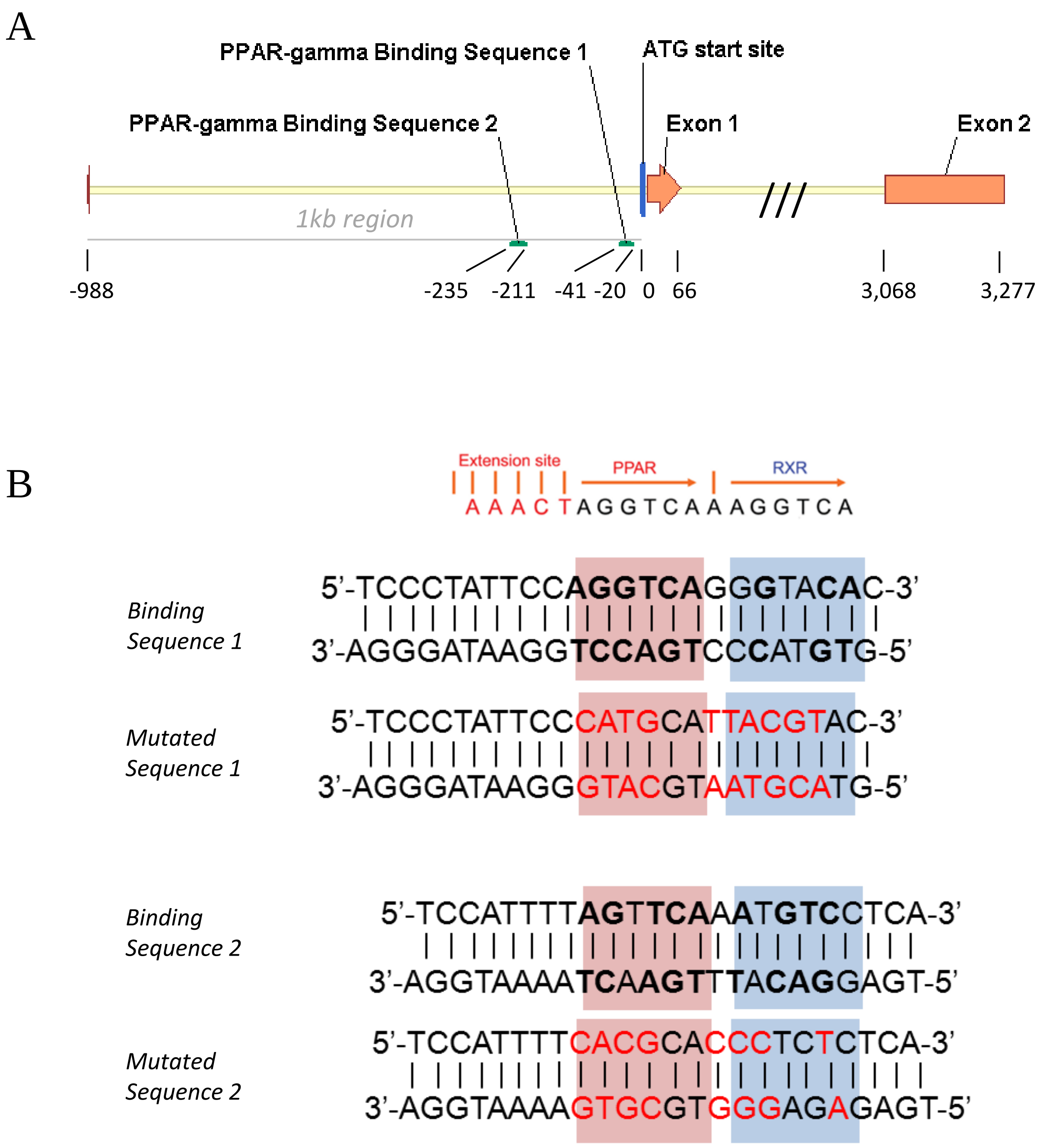

Supplementary Figure 1. PPAR-γ response element (PPRE) identification and binding and mutated sequence design. (A) Gene map of the 1kb upstream region of the human *GCM-1*. The 1kb region upstream of the human *GCM-1* gene was analyzed for PPAR-γ binding sites. Two putative PPREs were found within 250 base pairs from the transcription start site (sequence 1 and sequence 2). The gene map shows only exons 1 and 2 of *GCM-1* gene (not to scale). **(B)** Duplex oligonucleotide sequences for binding and mutated sequence 1 (top) and binding and mutated sequence 2 (bottom). Nucleotides conserved between the consensus PPRE (shown above for comparison) and our identified binding sites are bolded. Mutated nucleotides are shown in red. PPAR-γ binding site is highlighted in pink, RXR-α binding site shown in blue.

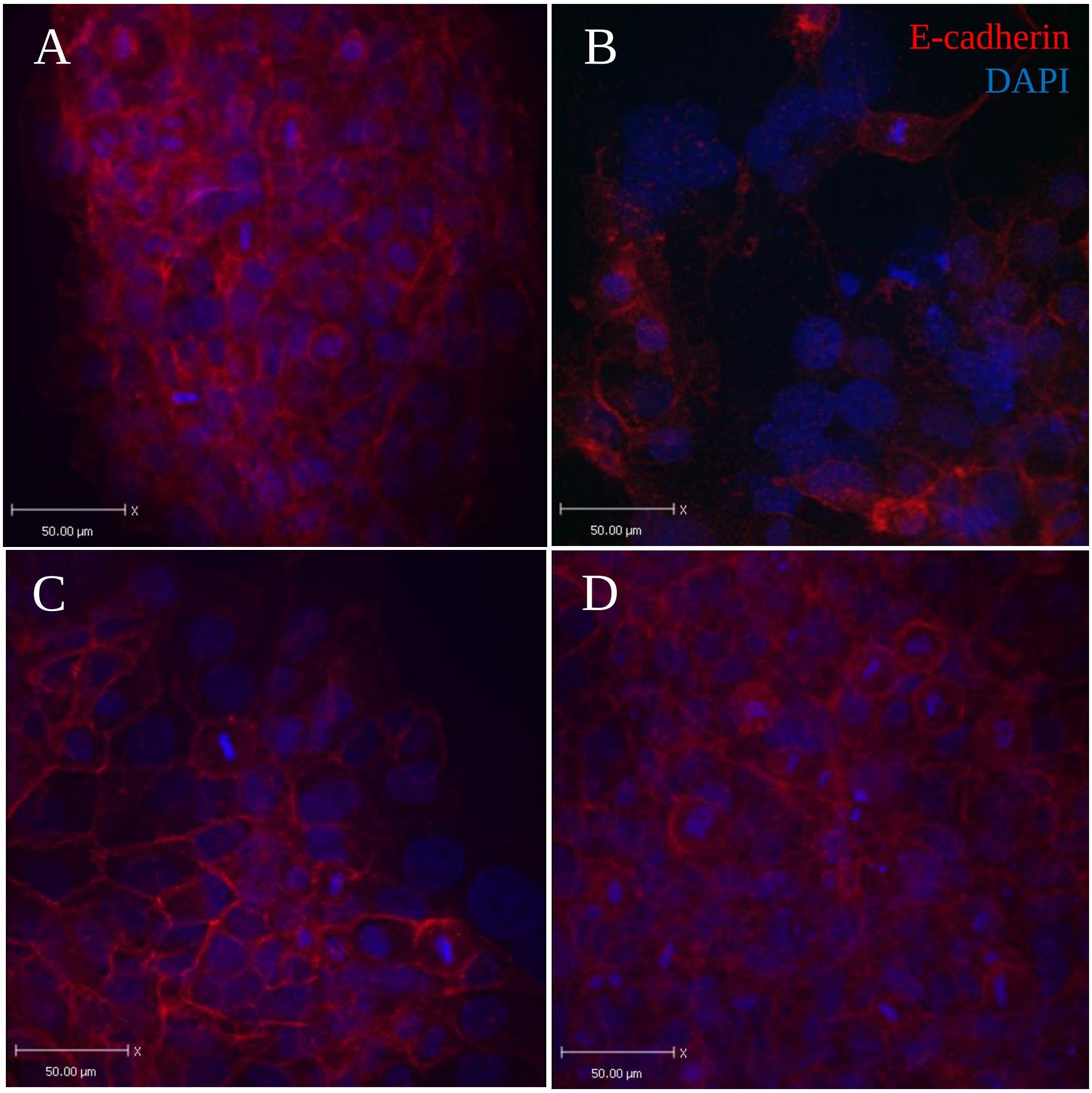

Supplementary Figure 2. Effect of PPAR-γ modulation on BeWo cell fusion. Cell fusion was assessed at 48 hours post-treatment. Cells were treated with forskolin (**B**) as a positive cell-fusion control, rosiglitazone (**C**), and T0070907 (**D**) and compared to vehicle control (**A**). E-cadherin (red) was used as a cell surface marker; DAPI (blue) was used as the nuclear stain (200X magnification).
